# Perceptions, barriers, and challenges of oral care among nursing assistants in the intensive care unit: a qualitative study

**DOI:** 10.1186/s12903-024-03979-3

**Published:** 2024-02-14

**Authors:** Xingru Li, Lin Yao, Xinchen Yang, Meixia Huang, Bo Zhang, Tao Yu, Yun Tang

**Affiliations:** 1 Department of Neurosurgery, The First Affiliated Hospital of Wannan Medical College, Wuhu City, Anhui Province China; 2 School of Clinical Medicine, Soochow University, Suzhou, Jiangsu Province China; 3 Department of Nursing, Nanchang University, Nanchang City, Jiangxi Province China; 4 Department of Respiratory and Critical Care Medicine, The First Affiliated Hospital of Wannan Medical College, Wuhu City, Anhui Province China

**Keywords:** Intensive care unit, Nursing assistant, Oral care, Qualitative research, Rehabilitation

## Abstract

**Background:**

Although oral hygiene is closely related to various diseases, it is sub-optimal in the Intensive Care Unit (ICU). Oral care in the ICU is challenged by nursing workloads, low staffing, and higher acuity patients, there are few policies and written guidelines for oral care. Nurses often delegate oral care to nursing assistants (NAs) whose role is overlooked. This study is to explore the perspectives, obstacles, and challenges of NAs in the oral care of the ICU.

**Methods:**

A qualitative study and semi-structured interviews were conducted with NAs in three ICU units, and Colaizzi’s phenomenological method was used to analyze the records.

**Results:**

Initially, 13 NAs met the inclusion criteria, and two did not participate in this study as they refused to be recorded. Finally, 11 ICU NAs were interviewed, with three receiving face-to-face interviews and eight receiving telephone interviews. Using Colaizzi’s phenomenological method, two themes and eight subthemes emerged from the data, we examined the self-perception, barriers and challenges of NAs regarding oral care and identified the subthemes: (1) The target audience, frequency, and importance; (2) Role; (3) Evaluation; (4) Patient-related factors; (5) Oral care tools; (6) Psychology of NAs; (7) Lack of education and training; (8) Lack of team support.

**Conclusion:**

Nursing assistants whose roles are overlooked by the nursing team are important members of the ICU team. Though oral care is closely related to disease prevention, it is rarely considered an essential task. Major barriers to implementing oral care in the ICU environment and patients include the psychological quality of participants, non-standard education and training, and inadequate team support. The expectation is that medical personnel will prioritize oral hygiene and recognize the significance of NAs in nursing work. Furthermore, future ICU oral care should investigate suitable tools and mouthwashes, simplified and standardized processes, standardized training, and multidisciplinary team collaboration.

**Supplementary Information:**

The online version contains supplementary material available at 10.1186/s12903-024-03979-3.

## Background

Intensive Care Unit (ICU) patients generally have critical conditions with a long course of disease, with most of them having low self-care ability or even losing their self-care ability. The conditions may worsen when general patients are admitted to the ICU, which may be due to the use of tracheal intubation, nasal feeding, sedatives, and a lack of salivary secretion that alters the oral environment [1]. A study showed that 92% of patients experienced oral changes during their ICU stay, often manifested as chapped lips, pale tongue coating mucosa, candidiasis etc. [2].. Oral care is the practice of assessing and caring for an individual’s oral cavity to prevent, manage, or eliminate oral disease [3]. Improving oral hygiene of ICU patients can reduce hospital-related infections, and high-quality oral care can minimize ventilator-associated pneumonia by 33.3% [4, 5]. Oral care is an essential hygiene requirement for every ICU patient, whether intubated or not. Appropriate oral care counteracts discomfort caused by xerostomia, oral pain or lip ulcers and promotes oral health by preventing dental caries and decay, bacterial or candidal stomatitis, gingivitis and periodontitis, which is associated with systemic diseases such as bacteremia, rheumatoid arthritis and cardiovascular disease [6]. However, most routine interventions in the ICU are limited to fundamental nursing tasks, and oral health concerns are frequently neglected [7, 8]. Variability in individual practices, lack of emphasis, disparities in educational and professional contexts, low oral care prioritization, and perceived lack of benefit in clinical practice may all contribute to this [1, 9].

Currently, only a few countries have oral care-related guidelines and agreements. A guideline in South Korea suggested that oral care for ICU tracheal intubation patients should be evaluated according to high-risk patient guidelines [10]. In ICU, oral care is challenged by nursing workloads, low staffing, and higher acuity patients. The ICU nursing team comprises professional nurses and unlicensed assistive personnel, such as nursing assistants (NAs). They can assist with many patient-care tasks, such as personal and oral care, which greatly support the healthcare team [11]. 55.7% of nurses delegated oral care to NAs [12]. A growing number of studies focus on the oral health status of ICU patients, but relatively few investigate the perspectives and challenges of NAs in the oral care of ICU patients. Therefore, this study supplements this gap through qualitative research.

The following research questions guided this study:

1. What are the ICU NAs’ perspectives and experiences of oral care?
2. What are the difficulties and challenges encountered by ICU NAs in implementing oral care?

## Method

### Study aims

1. To understand the current situation of oral care implementation for ICU patients.
2. To explore the perspectives, barriers, and challenges of oral care for NAs in the ICU.

### Design

This study strictly followed the guidelines of qualitative research [13]. The research used the descriptive phenomenology method which was developed on Husserl’s phenomenological theory [14], emphasizing returning to things themselves, aiming at depicting the real world, making people listen to phenomena fully and truthfully, and learn to acquire knowledge from their direct perception, observation and intuition.

### Sample

NAs were eligible for the following criteria: (1) Age >18 years old; (2) Working time ≥ 6 months; (3) Oral care was included in the nursing task. Participants who did not agree to be interviewed or recorded were excluded. Informed consent forms were emailed or in person to participants who met the criteria. In this study, four NAs were selected through purposive sampling, with other participants recruited based on the snowball method. The interviewer sought out the next potential participant by asking “Do you know of any other ICU NAs who perform oral care at this hospital?” The interviews were conducted only once, participant recruitment continued until saturation was reached.

### Data collection

Participants took part in interviews with the interviewer, who had worked in the ICU from another hospital, held a postgraduate degree in nursing, and had an interest in qualitative research without prior association with the participant. Before the interviews, an interview outline was developed through group discussion based on the research objectives and literature review, with the panel composed of professionals with experience in nursing, clinical medicine, dental specialties, and qualitative research). The interviewer underwent training from experts in qualitative research and two NAs were selected for the pilot. The interview outline was adjusted based on the feedback. The main interview questions are as follows: Q1: What is your understanding of oral care? Q2: What difficulties have you encountered in the process of oral care? Q3: What training have you received in oral care? Q4: What are your suggestions for oral care for ICU patients? The final detailed version can be found in Appendix 1. The interview was conducted in a quiet room with one participant seated in the conversation room, three in the meeting room, and the remaining participants on the phone in a calm environment. The topic of the interview was about the experience, barriers, and challenges of the NAs in the oral care of ICU patients. All interviews were recorded, noted, and transcribed and each of them lasted between 30 and 50 min.

### Analysis

A qualitative data analysis computer software, NVivo, was used for coding. This study adopted the Colaizzi phenomenological analysis method, which comprises the following seven steps: (1) Thoroughly record and peruse all interview materials; (2) Extract meaningful and consistent statements from the research phenomenon; (3) Summarize and extract meaning from meaningful statements; (4) Search for common characteristics or concepts of meaning, forming themes, theme groups, and categories; (5) Establish a connection between the identified themes and the research phenomenon and provide a complete narrative; (6) Integrate the obtained results, provide a detailed description of the research phenomenon, state the essential structure that constitutes the phenomenon, and provide feedback to the research subjects for confirmation to improve the validity of the study. During the verification process, if new information is generated, it is necessary to integrate them into a detailed description. The analysis was conducted concurrently with data collection so that data saturation could be evaluated. Data saturation was assessed and agreed upon by the research team on an ongoing basis.

### Rigor

The trustworthiness and rigor were achieved by ensuring dependability, confirmability, credibility and transferability of data [15]. Credibility was enhanced by using the guidelines of qualitative research to ensure a consistent approach to interviewing. Reliability and confirmability were achieved by establishing recordings, translations, and collating data back to the research subject for verification. We presented sufficient raw data in the form of direct quotes and a detailed process of the research to enable readers to evaluate the results and assess transferability to other settings and populations.

## Results

Data saturation was achieved after the 11th ICU NA was interviewed. In total, nine women and two men aged 21−34 years old were interviewed. Two participants received undergraduate education. Two participants are from urban areas, while the remaining were from rural areas. Their working years ranged from six months to six years, with two participants having experience working in dental clinics. Finally, four NAs participated in face-to-face interviews, while the remaining were interviewed via telephone. Table 1 depicted the general information and codes of participants.

**Table 1:** Characteristics of participants

Code	Gender	Age (years)	Educational background	Duration of service(years)	Residence	Working experience in a dental hospital or clinic
S1	F	24	U	0.5	R	N
S2	F	25	J	1	R	N
S3	F	34	U	15	R	N
S4	M	27	J	4	U	N
S5	M	23	J	1	R	N
S6	F	26	J	3	R	Y
S7	F	27	J	1	R	N
S8	F	24	J	1	R	N
S9	F	30	J	6	U	N
S10	F	21	J	1	R	Y
S11	F	25	J	0.5	R	N

Note: F: female; M: male; J: Junior college education; U: Undergraduate degree; U: urban; R: rural; Y: Has work experience in a dental hospital or clinic; N: Lack of work experience in dental hospitals or clinics

### Findings

Two themes and eight subthemes have emerged from the data (Table 2).

**Table 2:** Major themes and sub-themes

Theme	Sub-theme
Self-perception toward oral care	The target audience, frequency, and importance
	Role
	Evaluation
Barriers and challenges	Patient-related factors
	Oral care tools
	Psychology of nursing assistant
	Lack of education and training
	Lack of team support

### Self-perception toward oral care

Diverse perspectives on oral care among ICU NAs resulted from differences in education, professional experience, site of employment, and types of illnesses. During our discussion, the following subjects predominated:

### The target audience, frequency, and importance

The oral cavity should be evaluated in all critically ill patients [16]. Most NAs thought that patients with coma, inability to eat, high fever, and endotracheal intubation needed oral care; only two caregivers with experience in dental clinics reported that “everyone needs oral care.” [S6, S10] Some NAs described their views on the frequency of oral care in the ICU,

“I think, normally, once each morning, noon, and evening is enough; there is no need to do it twice during the day.” [S2].

Certain coma patients, including those who have suffered traumatic brain injury, must passively tolerate the frequency of brushing. Patients reported that excessive oral nursing frequency impacted their sleep quality. A NA expressed that,

“Normal people do not need [four times a day], but they [unconscious patients] cannot do it. So if they do it a little more often, it would not be a big problem.” [S8].

“Patients who are awake have more questions, like, ‘Why am I still doing oral care at this time of night? I am going to sleep.‘“[S9].

The guideline committee considered that oral care for critically ill patients should be done at least twice a day [17]. Lack of standardized training and evidence-based programs in oral care contributes to the variation in the frequency of oral care. Each participant talked about the benefits of oral care, such as keeping the mouth clean, preventing fungal infections, and improving appetite. The benefits of oral care also include preventing rheumatoid arthritis, cerebrovascular diseases, and ventilator-associated pneumonia; however, the relation between oral hygiene and the conditions above has yet to be formally investigated. The participants often expressed like this: “[Hesitating], um… I don’t know.’’ [S1]. The same goes for the NAs with long years of working experience, with phrases like “Hmm…” [S9], “This…” [S6]. Only one NA with a background in dental clinical work gave a brief answer, “I only know about oral cancer, but the rest…” [S3].

The workload in the ICU is heavy, and limited time and insufficient human resources make oral care easily neglected, whether by patients, doctors, nurses, or NAs. Some participants stated that oral care was a simple and basic task with almost no difficulty; therefore, few considered it the top priority of their work tasks.

“Basic, special basic, [everyone] can do it, how difficult is it?” [S1].

“In the ICU, [oral care] is essential because the patients cannot take care of themselves, and basic care is essential, outside [the ward], may… If they could care for yourself, there might be no need…” [S8].

Oral care is an essential part of both the ICU and the ward. Most NAs defined their nursing tasks as temperature measurement, nebulization, oral care, and bathing. Other participants, except the NA mentioned previously, believed oral care for ICU patients was insignificant. This difference was primarily attributable to the frequent emergencies that occurred in the ICU. They cannot proactively provide oral care unrelated to the patient’s life in the ICU.

### Role

NAs may come from other institutions without specialized education or corresponding certificates, and there was a clear division of labor between them and professional nursing staff. Almost all participants cannot participate in oral care for tracheal intubation patients as they mainly assisted bed nurses in fixing endotracheal intubation.

“[This]……[patients] who with endotracheal intubation are all done by teachers, and sometimes we will hold this intubation by the side, and we have never done it.” [S3].

Some participants stated that the bed nurse should complete health education on admission and discharge, and the bed doctor should assess patients’ oral condition.

“Nurses rarely do it [carry out health education] with their families; bed nurses complete this task, and we rarely talk to them.” [S3].

“Evaluating patients is not what we do.” [S5].

Oral care for critically ill patients should not just be the responsibility of doctors and nurses. The oral health team should be comprised of multiple providers (nurses, nursing assistants, physicians, dentists, dental assistants, dietitians, respiratory therapists, speech-language pathologists, etc.) working together to implement a comprehensive and individualized oral care program.

### Evaluation

Oral care assessment is crucial before entering the formal process, allowing for a targeted selection of personalized tools and mouthwashes. The most common method for evaluating oral care is to use a tongue depressor and a flashlight for bedside observation. However, there is a lack of evaluation of patients during admission and discharge, and obvious differences exist in evaluation methods and the required tools in the ICU, implying a lack of consensus on oral care assessment.

“If the patient can open their mouth, just look at the tongue coating and brush it. Is not the evaluation just a look?” [S1].

“Is there a denture in the mouth? Is there any damage to the mouth mucosa using a tongue depressor and a flashlight?” [S6].

“We do it [oral care] while evaluating; we all do it in a unified way, and all patients are the same.” [S4].

The assessment of oral care in critically ill patients should include the condition of the teeth, gums, tongue, mucus, membranes, and lips [17], but few NAs were able to do so. In addition to the above-mentioned participants, NAs with long working experience believed that the evaluation consisted of “oral smell, mucous membrane, tongue coating color, tooth mobility, dentures.” [S3, S9]. Observation is the method that is most frequently mentioned. Unexpectedly, neither the evaluation of objective instruments nor the assessment of oral care for special patients (e.g., those with swallowing disorders) were topics of discussion.

### Barriers and challenges

The challenges NAs faced in oral care mainly came from four aspects: patient-related factors, oral care tools, psychological factors, and social support.

### Patient-related factors

ICU patients were critically ill in a coma, and artificial airways, muscle rigidity, lockjaw, and agitation were the main reasons leading to the difficulty of NAs delivering oral care.

“Some patients are restless and will bite my cotton ball.” [S2].

“When you go to do it for him, he will bite very tightly. This kind of thing requires a tongue depressor, and he also shakes his head, so you can’t control him at all… The patient is irritable, he resists you, and you cannot even use a tongue depressor because he keeps moving and cannot put it in his mouth.” [S8].

Conscious patients have a higher level of cooperation than patients with blurred consciousness. They knew more about their oral conditions than NAs and asked to take oral care alone instead of being taken care of.

“Big cotton swabs are quite easy to use… It is very easy to use for awake patients, and it will be cleaner for patients to use them themselves than for us to wipe them by hand.” [S7].

However, some participants said, “He brushes himself just to pound it casually” [S10], and there were also patients companied with anxiety due to illness, which lead to a delay in oral care.

“[The patient] stayed in the ward for a long time and had emotional problems. At that time, [The patient] did not want to do it; we did not give him [oral care] when he is in a better mood in the afternoon, do it for him again.” [S5].

Some patients decline all oral care out of concern that they will lose their dentures, while others abandon treatment voluntarily.

“He does not want to… [They] are afraid that dentures will be lost; it is safest to keep them in his mouth.” [S10].

### Oral care tools

The oral care tools include sterilized oral bags, large cotton swabs, and suction tubes. Only two NAs reported using suction tubes, “Suction tubes were used on Monday.” [S7]. “I know we have them in our hospital, but not in our department.” [S1] Conscious patients were more willing to independently use a large cotton swab for oral care. Although the large cotton swab offers convenience in terms of portability, its delicate texture renders it susceptible to breakage and loss in the patient’s oral cavity. The oral bag includes curved forceps, straight forceps, tongue depressors, curved discs, and cotton balls. The unpacking and placing of the bag are cumbersome, but the safety index is high.

“It may be that the preparation time is relatively long because when you open the oral bag, you have to take out the bowl, then the pliers and tongue depressor should be distributed one by one, then cotton balls should be added, and the mouthwash should be poured into it. This step is a bit long.” [S7].

“The oral bag distributed uniformly in the hospital… is not very convenient, but it is relatively safe. When I used to work, I used large cotton swabs, which were more convenient, but there were certain safety hazards because they were not sturdy and could break off.” [S4].

Furthermore, mouthwashes containing chlorhexidine and hydrogen peroxide were frequently mentioned. Several NAs reported that patients refused oral care due to the bitter taste of mouthwash.

“They [mouthwash] are very bitter; I have rinsed [chlorhexidine]; many patients don’t like this taste.” [S7].

Both mouth packs and large cotton balls have their pros and cons. The foam rod may be immersed in the liquid for a long time, affecting the adhesion strength between the foam and the rod, thus increasing the potential suffocation risk, combining the two for oral care was recommended by nursing assistants. Furthermore, improving the taste of mouthwash is expected to increase patient compliance.

### Psychology of nursing assistant

The primary concerns of NAs are the potential degradation of the patient’s tracheal intubation and the misplacement of oral tampons. Furthermore, certain NAs have reported that unexpected sputum ejection of patients caused involuntary retraction of their bodies.

“I am afraid that some patients may spit, drool, or have cotton balls lost in their mouths.” [S6].

“[I] fear the tube being pulled out, causing the patient to suffocate.” [S2].

### Lack of education and training

Oral care lacks systematic training and professional guidance. Internet, official accounts, books, and bedside education are the most important ways to acquire oral care knowledge.

Most NAs indicated that oral care ‘training’ had only been conducted at the time of entry; after employment, the ‘training’ was performed through bedside teaching. Some participants stated that the oral care procedures mentioned in books took a long time and were unsuitable for critically ill patients as only a few people can follow the book for oral care, whereas most will follow their ideas to save time for nursing tasks that they consider important. Furthermore, the ICU has heavy tasks and a lack of human resources, which urges nursing assistants to speed up the operation process of oral care.

“It is not very standard. I will keep wiping until it is clean according to the patient’s needs.” [S7].

“On the night shift, we do oral care for all patients by ourselves. Sometimes, the beds are full. If a person follows the [book] order, we cannot finish it in an hour, and thus, one should speed up the process.” [S8].

### Lack of team support

Oral care is primarily the responsibility of NAs and nurses. Surprisingly, few participants mentioned that doctors pay attention to patients’ oral care.

“Doctors generally do not pay attention to oral care unless there is some damage, and then they will pay attention to it.” [S11].

“Some nurses might say that a certain patient has a foul mouth and needs to be brushed with hydrogen peroxide, but the doctor… did not hear much about it.” [S8].

ICU patients may face life-threatening situations at any time. Usually, doctors focus on disease treatment, while oral care appears insignificant. Moreover, professional nurses mostly conducted health education for patients upon admission and discharge, with little mention of oral care. Doctors and NAs were not involved in this health education process.

## Discussion

Oral care has become indispensable in the ICU, whether an artificial airway or not, affecting patients’ general health and prognosis [18]. Current evidence suggests oral health often deteriorates throughout a patient’s hospital stay. Unfortunately, this is frequently the case in a critical care setting [19]. The reason is that oral care is often not considered a high priority during hospitalization, whether a doctor, nurse, NAs, or linguist, consistent with the present research [20]. In this context, we attributed multiple factors, such as the specificity of ICU patients, the advantages and disadvantages of nursing tools, NAs, and patient-related factors. Tanguay’s study also reported similar challenges [21].

Regarding oral care, numerous studies have documented the perspectives of doctors, nurses, and patients [22, 23]. Due to the unique nature of the ICU environment, the condition is critical, the tasks are heavy, and NAs play an important role in oral care [11, 24, 25]. This study conducted interviews with ICU NAs to understand the status quo of ICU oral care implementation, the difficulties and challenges faced by ICU NAs during oral care, and to provide basic data for examining the current status of oral care for critically ill patients and seeking ways to improve it. At the same time, this study can stimulate medical workers to pay attention to the role of NAs, promote the establishment of a multidisciplinary group of ICU oral care team, further promote the recovery of critically ill patients, reduce the incidence of complications, alleviate economic burdens, and eventually improve the quality of life.

It was proved that oral hygiene reduces the occurrence of pneumonia and accelerates recovery [26]. Unexpectedly, oral hygiene did not occupy the top three of NAs’ responsibilities, and they were not aware of the connection between oral hygiene and associated diseases, this is consistent with the study by Ab Malik [27]. Similarly, only 38% of nursing staff have experienced substantial training, according to Croft [1]. The role of oral bacteria in pneumonia and respiratory infections is well known [19], and unfortunately, no one has commented on the evaluation of oral care for special patients, such as those with swallowing disorders VAP, nor did anyone discuss the evaluation methods of objective tools. Comparatively, NAs with experience in dental clinics and those with seniority were more comprehensive and specific in oral assessment, implying that these two elements appear to be favorable factors for better implementation of oral care. Future quantitative studies could continue to explore the factors influencing oral health assessment.

Oral care intervention should be based on individual needs of patients. Therefore, detailed oral assessment should be carried out before oral care to provide patients with a targeted personalized oral care plan. Appropriate brushing tools and mouthwashes can promote oral care, although chlorhexidine has been proven effective [19], its taste should be considered in the future. It is well known that providing oral care for ICU patients is challenging and time-consuming [28]. Therefore, an oral assessment before providing oral care may be clinically and economically beneficial to the ICU [6].

The variability of oral care frequency and tools implies a need for more unified norms and processes. Researchers investigated the attitude and knowledge of nursing staff in the ICU of cardiothoracic surgery [1]. It was found that nursing staff had limited time, and oral care became a neglected field; more than two-thirds of them needed more standardized training. In a previous study [10], over 50% of nurses noted the need for expert guidance and practice. Although their research focus was on registered nurses, while our focus was on NAs, the results of the report were consistent. Researchers also explored oral care by caregivers and related healthcare providers, including NAs [29]. They pointed out that NAs from other services who may not have received corresponding education must be the members of oral care training. Professional education can help NAs overcome the phobia of cotton balls slipping off or moving catheters [30]. In our study, only two participants received undergraduate education, and the vast majority of NAs were from urban areas. Still, the NAs with varying levels of education and different places of residence did not report discernible differences in their perceptions of oral care delivery. Most NAs had no oral care experience, and although NAs with experience working in a dental clinic showed a more comprehensive understanding of oral care and cited the importance of regular dental check-ups, they were largely unaware of the relationship between oral care and disease.

Given that nursing staff are the major healthcare providers responsible for maintaining oral health, the absence of specific didactics in this area represents a focus for future research and quality improvement. Future research will be required to develop and execute interprofessional dental care training programs and assess their impact on nurse competencies and patient outcomes.

Establishing a multidisciplinary team for oral care training should become a future trend [31]. Hammond et al [32] emphasized multidisciplinary cooperation in their research. However, the present study found doctors rarely participated in oral care training in the ICU. Many researchers have pointed out the importance of dental professionals providing professional guidance on oral care for ICU patients [10, 12]. The future comprehensive oral care plan should involve nurses, nursing assistants, occupational therapists, dental experts, language medical record holders, nutritionists, and rehabilitation therapists [33]. So far, there are few policies and written guidelines for the oral care of ICU patients. Despite these written guidelines, the current utilization of oral care guidelines is very low, and future research still needs to explore the best evidence-based guidelines.

This study has the following limitations: First, the scope of this qualitative interview was confined to a single institution, thereby constraining the applicability of the study; Secondly, the working age of the participants is relatively short, and the reliability of the results is not guaranteed; Finally, most of the research is conducted through telephone interviews, and future research can further enhance the credibility of the data.

## Conclusion

Oral care is closely related to disease prevention, yet NAs rarely consider it a vital responsibility. Furthermore, standardizing the assessment and frequency of oral care is essential. NAs serve as an important part of the healthcare team, but their role in oral care is overlooked. The uniqueness of the ICU setting and patients, the psychological quality of participants, sub-standard education and training, and inadequate team support are major challenges to their oral care implementation. Moreover, suitable tools and mouthwashes, streamlined and standardized processes, proper training, and multidisciplinary team collaboration are areas worth exploring in future ICU oral care.

### Electronic supplementary material

Below is the link to the electronic supplementary material.

Supplementary Material 1

## Data Availability

All data generated or analyzed during this study are included in this published article and its supplementary information files.

## Abbreviations

ICU: Intensive Care Unit
NA: Nursing assistants

## References

[1] Croft K, Dallal-York J, Miller S, Anderson A, Donohue C, Jeng E (2023). Provision of oral care in the cardiothoracic intensive care unit: survey of nursing staff training, confidence, methods, attitudes, and Perceived barriers. J Contin Educ Nurs.

[2] Quintanilha R, de Pereira MC, Oliveira MRR, de Penoni SP, Salgado DC, Agostini DR. M, Oral clinical findings and intensive care unit prognostic scores. BMJ Support Palliat Care. 2023;:spcare-2023-004479. 10.1136/spcare-2023-004479 37500568

[3] Bonetti D, Hampson V, Queen K, Kirk D, Clarkson J, Young L. Improving oral hygiene for patients. Nurs Stand R Coll Nurs G B. 1987. 2015;29:44–50. 10.7748/ns.29.19.44.e9383 25563127

[4] Lei S, Liu Y, Zhang E, Liu C, Wang J, Yang L (2023). Influence of oral comprehensive nursing intervention on mechanically ventilated patients in ICU: a randimized controlled study. BMC Nurs.

[5] Quinton K, Guy-Frank CJ, Syed S, Klugh JM, Dhanani NH, Adibi SS (2023). Poor oral health in Trauma Intensive Care Unit patients: application of a novel oral health score. Surg Infect.

[6] Labeau SO, Conoscenti E, Blot SI (2021). Less daily oral hygiene is more in the ICU: not sure. Intensive Care Med.

[7] Isac C, Samson HR, John A (2021). Prevention of VAP: endless evolving evidences-systematic literature review. Nurs Forum (Auckl).

[8] Li Y, Liu C, Xiao W, Song T, Wang S (2020). Risk factors, and outcomes of Ventilator-Associated Pneumonia in Traumatic Brain Injury: a Meta-analysis. Neurocrit Care.

[9] Klompas M (2010). Ventilator-associated pneumonia: is zero possible?. Clin Infect Dis off Publ Infect Dis Soc Am.

[10] Kim Y, Ku H-M, Jun M-K (2023). Knowledge evaluation of oral diseases and Perception of Cooperation with Dental experts for oral care of nurses in Intensive Care Units in Korea: a preliminary study. Nurs Rep Pavia Italy.

[11] Schneider M, Good S, Dowd M, Feil D (2023). How to help nursing assistants feel valued. Nurs (Lond).

[12] Arkia M, Rezaei J, Salari N, Vaziri S, Abdi A (2023). Oral status and affecting factors in Iranian ICU patients: a cross-sectional study. BMC Oral Health.

[13] Malterud K (2001). Qualitative research: standards, challenges, and guidelines. Lancet Lond Engl.

[14] Goretti S, Esposito CM, Di Petta G (2023). Phenomenology of psychiatric emergencies. Front Psychol.

[15] Krefting L (1991). Rigor in qualitative research: the assessment of trustworthiness. Am J Occup Ther off Publ Am Occup Ther Assoc.

[16] Collins T, Plowright C, Gibson V, Stayt L, Clarke S, Caisley J (2021). British Association of Critical Care Nurses: evidence-based consensus paper for oral care within adult critical care units. Nurs Crit Care.

[17] Berry AM, Davidson PM, Nicholson L, Pasqualotto C, Rolls K (2011). Consensus based clinical guideline for oral hygiene in the critically ill. Intensive Crit Care Nurs.

[18] Steinle EC, Pinesso JAM, Bellançon LB, de Paula Ramos S, Seixas GF (2023). The association of oral health with length of stay and mortality in the intensive care unit. Clin Oral Investig.

[19] Kelly N, Blackwood B, Credland N, Stayt L, Causey C, Winning L (2023). Oral health care in adult intensive care units: a national point prevalence study. Nurs Crit Care.

[20] Chipps E, Gatens C, Genter L, Musto M, Dubis-Bohn A, Gliemmo M (2014). Pilot study of an oral care protocol on poststroke survivors. Rehabil Nurs off J Assoc Rehabil Nurses.

[21] Tanguay A, LeMay S, Reeves I, Gosselin É, St-Cyr-Tribble D (2020). Factors influencing oral care in intubated intensive care patients. Nurs Crit Care.

[22] Curtin C, Barrett A, Burke FM, McKenna G, Healy L, Hayes M. Exploring facilitators and barriers associated with oral care for inpatients with dysphagia post-stroke. Gerodontology. 2023. 10.1111/ger.12709. 10.1111/ger.12709 37531498

[23] Horne M, McCracken G, Walls A, Tyrrell PJ, Smith CJ (2015). Organisation, practice and experiences of mouth hygiene in stroke unit care: a mixed-methods study. J Clin Nurs.

[24] Bellury L, Hodges H, Camp A, Aduddell K (2016). Teamwork in Acute Care: perceptions of essential but unheard assistive personnel and the counterpoint of perceptions of registered nurses. Res Nurs Health.

[25] Gent PL, Proulx JR, Seidl K (2014). The forgotten rung: a clinical ladder for UAP. Nurs Manag (Harrow).

[26] Wu C, Huang H, Xu W, Li J, Chen M, Zhao Q (2023). Influencing factors associated with oral health among older hospitalized patients with ischemic stroke: a cross-sectional survey. Int J Nurs Sci.

[27] Ab Malik N, Mohamad Yatim S, Hussein N, Mohamad H, McGrath C (2018). Oral hygiene practices and knowledge among stroke-care nurses: a multicentre cross-sectional study. J Clin Nurs.

[28] Grap MJ, Munro CL, Ashtiani B, Bryant S (2003). Oral care interventions in critical care: frequency and documentation. Am J Crit Care off Publ Am Assoc Crit-Care Nurses.

[29] Ferguson C, George A, Villarosa AR, Kong AC, Bhole S, Ajwani S (2020). Exploring nursing and allied health perspectives of quality oral care after stroke: a qualitative study. Eur J Cardiovasc Nurs.

[30] Ames NJ (2011). Evidence to support tooth brushing in critically ill patients. Am J Crit Care off Publ Am Assoc Crit-Care Nurses.

[31] Nowghani F, Lisiecka D, Phelan S, Horan P, O’Reilly L, Howell Y, et al. Keep my teeth: an evaluation of multi-disciplinary training in mouth care for people with intellectual developmental disorders. Spec Care Dent off Publ Am Assoc Hosp Dent Acad Dent Handicap Am Soc Geriatr Dent. 2023. 10.1111/scd.12927. 10.1111/scd.12927 PMC13193427 37779096

[32] Hammond L, Conroy T, Murray J (2023). Exploring oral care practices, barriers, and facilitators in an inpatient stroke unit: a thematic analysis. Disabil Rehabil.

[33] Murray J, Scholten I (2018). An oral hygiene protocol improves oral health for patients in inpatient stroke rehabilitation. Gerodontology.

