# Supplementary material for: Perceptions, barriers, and challenges of oral care among nursing assistants in the intensive care unit: a qualitative study
Source: BMC Oral Health. 2024 Feb 14;24:235. doi: 10.1186/s12903-024-03979-3 (PMC10868102; doi:10.1186/s12903-024-03979-3)
Supplement: Supplementary file 1 — Supplementary Material 1 [file 12903_2024_3979_MOESM1_ESM.docx]

**Attachment: Outline of Qualitative Interview**

Q1: What is your understanding of oral care?

1-1 Who do you think needs oral care in ICU？

1-2 How often do you implement oral care?

1-3 What do you think are the benefits of oral care?

1-4 What is the position of oral care in your mind?

Q2: What difficulties have you encountered in the process of oral care?

2-1 What do you think about oral care tools?

2-2 What is the difference between conscious patients and unconscious patients?

Q3: What training have you received in oral care?

3-1 Where do you usually obtain knowledge about oral care?

3-2 What is the basis for implementing oral care?

Q4: What are your suggestions for oral care for ICU patients?
